# Measuring indirect effects of rotavirus vaccine in low income countries

**DOI:** 10.1016/j.vaccine.2016.07.001

**Published:** 2016-08-17

**Authors:** Aisleen Bennett, Naor Bar-Zeev, Nigel A. Cunliffe

**Affiliations:** Malawi-Liverpool-Wellcome Trust Clinical Research Programme, P.O Box 30960, Chichiri, Blantyre 3, Malawi; Institute of Infection & Global Health, University of Liverpool, 8 West Derby Street, Liverpool L69 7BE, UK

**Keywords:** Rotavirus, Gastroenteritis, Vaccine, Herd effects

## Abstract

Widespread introduction of rotavirus vaccines has led to major reductions in the burden of rotavirus gastroenteritis worldwide. Vaccine effectiveness is diminished, however, in low income countries, that harbour the greatest burden of rotavirus attributed morbidity and mortality. Indirect effects of rotavirus vaccine (herd immunity and herd protection) could increase population level impact and improve vaccine cost effectiveness in such settings. While rotavirus vaccine indirect effects have been demonstrated in high and middle income countries, there are very little data from low income countries where force of infection, population structures and vaccine schedules differ. Targeted efforts to evaluate indirect effects of rotavirus vaccine in low income countries are required to understand the total impact of rotavirus vaccine on the global burden of rotavirus disease.

Prior to the global roll-out of rotavirus vaccines, rotavirus gastroenteritis was responsible for 450,000 under-5 deaths each year, over 95% of which occurred in GAVI Vaccine Alliance-eligible countries [1]. Clinical trials in high and middle income countries (HMIC) of two globally licensed, live oral rotavirus vaccines (a monovalent (RV1) and pentavalent (RV5) vaccine) demonstrated 85–100% efficacy against severe rotavirus gastroenteritis, that has translated into impressive effectiveness following their introduction into national childhood immunisation programmes [2]. In low income countries (LIC) vaccine efficacy was substantially lower (48–61%) [3], [4]; however in view of the high rotavirus gastroenteritis associated case fatality in these countries the WHO recommended routine implementation of rotavirus vaccine for infants as a global priority. Since 2012, rotavirus vaccine has been introduced into the infant immunisation schedules of more than 35 GAVI eligible countries, with two or three doses given in early infancy for RV1 and RV5 respectively. Encouraging post-introduction findings have recently been reported from sub-Saharan African countries with RV1 effectiveness of 64% in Malawi, and a 61–70% reduction in rotavirus attributable hospitalisations following introduction of RV5 in Rwanda [5], [6].

In addition to evaluating the direct effect of vaccines (protection afforded directly to the individual by the vaccine), it is important also to consider vaccine indirect effects when evaluating the total impact of vaccination programmes. Indirect effects describe reduction in disease burden due to changes in transmission resulting from vaccination [7]; they can occur both in unvaccinated individuals, and in addition to direct effects in vaccinated individuals [8]. Indirect effects of vaccines may result from horizontal transmission of vaccine leading to immune protection in unvaccinated contacts (herd immunity) or reduction in transmission of wild-type infection (herd protection) [9]. The latter may be generated via two mechanisms; a reduction in number of infected cases with subsequent reduced likelihood that susceptible community members will come into contact with an infectious individual; and through a reduction in the infectiousness of a vaccinated case if they do acquire disease [10]. Some vaccines, for example measles or rubella vaccines, provide herd protection only, while live, oral vaccines such as rotavirus and oral poliovirus vaccine (OPV) can provide both herd protection and herd immunity [9].

Indirect effects following rotavirus vaccination have been reported from Europe [11], [12], [13], the USA [14], Latin America [15] and Australasia [16], with a reduction in disease seen in unvaccinated young children, as well as vaccine age-ineligible groups including infants under 2 months of age [17], [18], and older children and adults [19]. A review of data from several HMIC estimated a median herd effect for infant rotavirus vaccine of 22% [20]. These effects have been derived by comparing observed to expected vaccine impact, or by measuring reductions in disease burden in those too old to have been vaccinated [11], [19], [20], [21]. Although the precise mechanisms of these observed indirect effects are not known, both herd protection and herd immunity have potential to contribute. In Taiwan, where qRT-PCR was used to detect rotavirus vaccine virus, shedding was detected in up to 90% of recipients of a single dose of either RV1 or RV5 on days 4–7 following vaccination [22]. Horizontal transmission of RV5 to siblings of vaccinated infants was described in the US [23] and transmission of RV1 occurred in 19% of 100 sets of twins during a randomised controlled trial in the Dominican Republic; approximately a quarter of whom developed anti-rotavirus IgA sero-conversion [24]. Regarding herd-protection, wild-type rotavirus transmission is associated with the presence of symptoms in the index case, and symptomatic infants are thought to be crucial in introducing rotavirus infection into households [25], [26]. Additionally, rotavirus vaccine protects maximally against severe disease, and severity of rotavirus diarrhoea has been shown to correlate with the quantity of virus shed in the stool and the risk of transmission [25], [27]. It is therefore plausible that vaccine-induced reduction in either clinical disease frequency or severity in young children might be expected to reduce transmission of rotavirus in the community, though definitive evidence of this is lacking.

As a result of the higher rotavirus disease burden and reduced vaccine effectiveness in LICs, the occurrence of rotavirus indirect vaccine effects would be particularly important, since additional vaccine benefit would increase total population impact and improve vaccine cost-effectiveness. This has been demonstrated clearly with cholera vaccine, where re-analysis of clinical trials to include evaluation of indirect effects showed that cholera vaccine was not only cost effective, but has potential to have a significant impact on the burden of endemic cholera [28], [29]. However, with the exception of recent observational data from Rwanda, there is a lack of published evidence of rotavirus indirect effects from LICs [6]. LICs differ from HMICs in burden of disease, presence of co-morbidities such as HIV and malnutrition, vaccine effectiveness, timing and coverage of vaccine schedules, and population structures. As a result it is uncertain whether and to what extent rotavirus indirect effects will occur in these settings.

Accurate evaluation of vaccine indirect effects can be challenging, and this is especially true in low resource environments. Indirect effects of vaccination programmes are typically estimated using observational data after vaccine introduction; including population level disease surveillance, sero-surveys or case control studies comparing odds of disease between contacts exposed to vaccinated or unvaccinated individuals. Indirect effects can be inferred if observed reductions in disease incidence are greater than those anticipated given vaccine coverage and effectiveness, but fully accounting for confounding in such observational studies can be difficult. In addition, many LICs lack the systematic surveillance systems required for adequate comparisons of disease rates among unvaccinated groups, and large scale sero-surveys can be problematic in settings where infra-structure is poor and sample collection is not always acceptable to local communities. Case-control studies can be difficult to conduct to a robust standard, particularly if laboratory confirmed endpoints are required.

Recently, study designs have been described which permit evaluation of indirect effects prospectively as part of clinical trials before vaccine introduction [30]. Such designs could include prospective cluster-randomised trials comparing disease incidence among unvaccinated individuals within communities randomised to receive vaccination or placebo, and step-wedge designs in which vaccination is sequentially introduced into clusters or regions allowing the as-yet unvaccinated cluster to act as a control [31]. Such studies represent an improvement on observational strategies as they allow quantitative measurement of indirect effects whilst minimising bias, but they are complex and expensive to undertake and in the case of rotavirus it is now unethical to randomise to placebo given recommendation for global introduction and the demonstrated effectiveness of vaccination. However there may still be scope for such studies, for example in situations where sub-national vaccine introduction is planned. Halloran et al. [32] have argued that small units such as households or family units can be used as an alternative to larger clusters, with disease rates of household contacts in vaccinated units being compared to those of household contacts in unvaccinated units. Such units can be used to investigate overall indirect effects, or more specific components of indirect effects such as vaccine effect on infectiousness or transmission of vaccine type virus from vaccinated infants to household members, and may be an efficient and pragmatic approach to evaluating vaccine indirect effects in low-resource settings [32].

All methods to evaluate vaccine indirect effects involve either assessment of disease burden before and after vaccine introduction, assessment of vaccine impact at the time vaccine is introduced, or the ability to assess a partially vaccinated community. As increasing numbers of countries introduce rotavirus vaccine into their routine infant schedules and achieve close to universal coverage the window of opportunity to evaluate rotavirus vaccine indirect effects in LICs is limited and it is important to ensure existing opportunities are not lost. International collaborations and triangulation of data across multiple sites and different study designs may be required. Mathematical models may be important to allow incorporation of heterogeneous data, and to predict the contribution of indirect effects to rotavirus vaccine total impact.

Rotavirus vaccine programmes have made a substantial impact on diarrhoeal disease in children in the poorest countries. However to support ongoing policy development, particularly for countries transitioning out of GAVI support, it is crucial that the overall population level effect of the vaccine is accounted for in assessments of impact and cost-effectiveness.

## Funding

This work was supported by a Wellcome Trust Programme Grant (Grant No. 091909/Z/10/Z) and the MLW Programme Core Award from the Wellcome Trust. AB was supported by a WT clinical Ph.D. fellowship (Grant No. 102466/Z/13/A).

## Conflict of Interest

NAC declares receipt of research grant support and honoraria for participation in rotavirus vaccine advisory board meetings from GlaxoSmithKline Biologicals. NBZ has received investigator initiated research grants from GlaxoSmithKline Biologicals and from Takeda Pharmaceuticals. AB declares no conflicts of interest.
